# Epigenetic regulation of cardiac physiology and pathophysiology: biological sex matters

**DOI:** 10.20517/jca.2025.36

**Published:** 2026-02-12

**Authors:** Omid M. T. Rouzbehani, Marta W. Szulik, Clint Gwynn, Sophie L. Stephens, Riley W. Porter, Isidoro Cobo, Manuel Rosa-Garrido, Sarah Franklin, Sihem Boudina

**Affiliations:** 1 Department of Nutrition and Integrative Physiology, Program in Molecular Medicine, University of Utah, Salt Lake City, UT 84112, USA.; 2 Nora Eccles Harrison Cardiovascular Research and Training Institute, University of Utah, Salt Lake City, UT 84112, USA.; 3 Division of Immunology and Rheumatology, The University of Alabama at Birmingham School of Medicine, Birmingham, AL 35294, USA.; 4 Department of Cellular and Molecular Signaling, The Institute of Biomedicine and Biotechnology of Cantabria, CSIC-University of Cantabria, Santander 39011, Spain.; 5 Department of Internal Medicine, Division of Cardiovascular Medicine, School of Medicine, University of Utah, Salt Lake City, UT 84112, USA.; 6 Department of Biomedical Engineering, The University of Alabama at Birmingham School of Medicine, Birmingham, AL 35294, USA.

**Keywords:** Epigenetics, histones, methylation, cardiovascular disease, biological sex

## Abstract

Cardiovascular disease (CVD) is the leading cause of global morbidity and mortality, with epigenetic mechanisms playing a pivotal role in its pathogenesis. This review synthesizes current evidence on sex-specific epigenetic regulation in cardiac health and disease, highlighting DNA methylation, histone modifications, and non-coding RNAs as key mediators. Epigenetic processes govern cardiac development, remodeling, and responses to injury, with sex chromosomes, sex hormones, and environmental factors contributing to dimorphic patterns. Developmental programming establishes early sex biases in chromatin architecture while aging and clonal hematopoiesis amplify these differences *via* mutations in epigenetic modulators. Therapeutic strategies targeting epigenetic regulators hold promise but require sex-tailored approaches to optimize efficacy and minimize off-target effects. This review underscores the critical need for sex-stratified research to advance precision medicine for CVD.

## Graphical Abstract

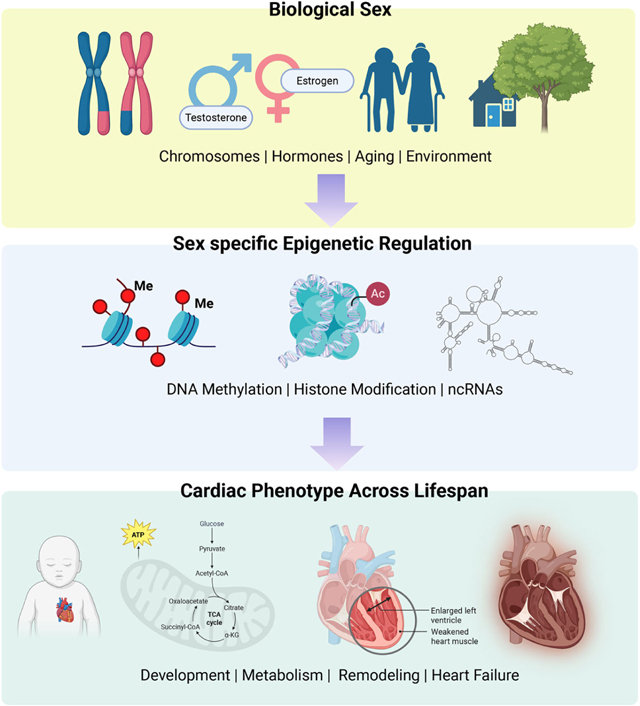

## INTORDUCTION

Cardiovascular disease (CVD) remains the leading cause of mortality worldwide, and its burden reflects not only traditional risk factors, including hypertension, diabetes, dyslipidemia, but also molecular regulatory layers that shape cardiac resilience and vulnerability. Emerging evidence highlights epigenetic regulation as a key determinant of cardiac health and disease trajectories^[1,2]^. Epigenetic mechanisms, including DNA methylation, histone modifications, chromatin remodeling, and noncoding RNAs, govern gene expression without altering the underlying DNA sequence, enabling dynamic and reversible regulation in response to developmental cues, environmental exposures, and pathological stress. In the heart, these mechanisms orchestrate key processes from embryonic cardiogenesis to postnatal maturation and adaptation to injury^[3,4]^.

Although the field of cardiovascular epigenetics has expanded rapidly, most studies have historically been conducted without considering sex as a biological variable. This omission overlooks a critical layer of complexity: men and women differ in cardiac structure, function, and disease presentation, with these differences shaped not only by sex chromosome composition but also by sex hormones and their downstream epigenetic consequences^[5]^. For example, sex-specific DNA methylation patterns, differential expression of histone-modifying enzymes encoded on the sex chromosomes [e.g., Lysine demethylase 6A (KDM6A) on the X chromosome and UTY (ubiquitously transcribed tetratricopeptide repeat-containing, Y-linked) on the Y chromosome], and hormone receptor-dependent recruitment of chromatin modifiers all contribute to distinct molecular landscapes in male and female hearts^[5–7]^. These differences can arise early in development and persist across the lifespan, shaping sex-specific susceptibility to and recovery from cardiovascular injury.

Recent advances in genome-wide profiling have revealed that epigenetic alterations underline numerous forms of cardiac pathophysiology, including heart failure (HF), ischemic heart disease (IHD), and cardiomyopathies^[3,8]^. Furthermore, emerging sex-specific findings suggest that the same epigenetic modification can exert divergent effects depending on biological sex, potentially explaining observed disparities in disease onset, severity, and therapeutic responses. Understanding these mechanisms has direct implications for precision medicine, where epigenetic targets could be leveraged to develop sex-specific diagnostic tools and treatments^[4,9]^.

**Figure 1.**
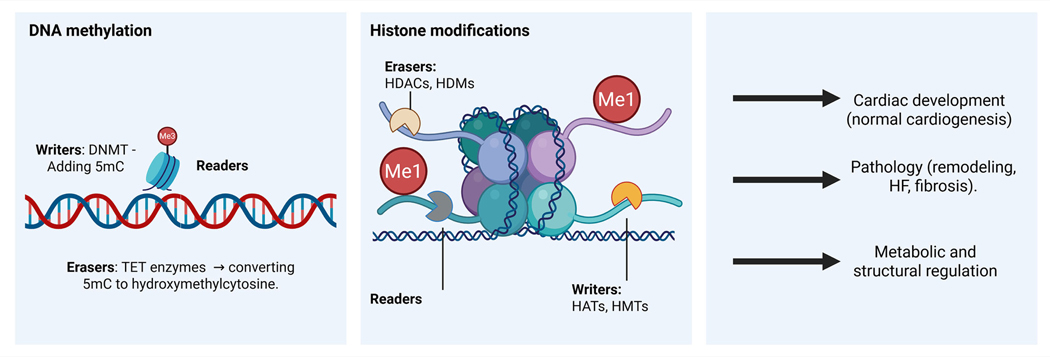
Epigenetic regulation of gene expression in the heart. DNA methylation, histone modifications, and chromatin remodeling act together to control cardiac development and adaptation to stress. DNMT: DNA methyltransferase; 5mC: 5-methylcytosine; TET: ten-eleven translocation (dioxygenase) enzymes; HDACs: histone deacetylases; HDMs: histone demethylases; HATs: histone acetyltransferases; HMTs: histone methyltransferases; Me1: mono-methylation; HF: heart failure. Figure created in BioRender. Stephens, S. (2026) https://BioRender.com/n26cejf.

In this review, we first provide an overview of fundamental epigenetic mechanisms operative in the heart, focusing on DNA methylation and histone modifications [Figure 1], and summarizing their roles in cardiac development, remodeling, and disease. We then discuss sex-specific epigenetic regulation and highlight its contribution to differences in cardiac physiology and pathophysiology. Finally, we discuss the therapeutic potential of targeting epigenetic pathways in a sex-conscious manner, emphasizing the need for future consideration of biological sex as a central variable in cardiovascular epigenetics research.

## BASICS OF EPIGENETIC MECHANISMS IN THE HEART

### DNA methylation

DNA methyltransferases (DNMTs) are epigenetic regulators that transfer a methyl group onto the C5 position of cytosine to form 5-methylcytosine (5mC), mainly in the context of cytosine phosphate guanine (CpG)^[10]^. DNA methylation typically promotes gene silencing by either directly blocking transcription factors from binding or by allowing binding of methyl-binding proteins and interacting with a co-repressor complex. Gene transcription can be activated by oxidation of 5mC by the ten-eleven translocation (TET) family of proteins^[11]^.

There are three DNA methylation enzymes: writers, readers, and erasers. Writers are the enzymes that catalyze methylation, including DNMT1, DNMT3A, and DNMT3B. DNMT3A and DNMT3B are *de novo* methyltransferases that add methyl groups to DNA, establishing the methylation pattern. DNMT1 is the maintenance DNMT, which adds methyl groups to cytosine residues left behind by prior methylation. This maintains the methylation pattern on DNA following replication. Eraser enzymes, such as TET proteins, remove methyl groups from previously methylated DNA, while reader enzymes, including the methyl-CpG binding domain (MBD) protein family, recognize methyl groups and influence transcription factor binding^[11]^.

### Histone modifications

Histone post-translational modifications (PTMs) regulate gene expression by altering chromatin accessibility and by recruiting transcriptional regulators^[12]^. These modifications occur primarily on the N-terminal histone tails and are dynamically controlled by writers (e.g., histone acetyltransferases (HATs) and methyltransferases (HMTs)), erasers (e.g., histone deacetylases (HDACs) and demethylases (HDMs)), and readers (e.g., bromodomains and plant homeodomain (PHD) fingers)^[13–15]^. Distinct histone variants are also deposited at specific genomic loci, where they help recruit regulatory enzymes and influence gene expression^[16]^. Specific PTMs, including acetylation, methylation, phosphorylation, ubiquitination and others, differentially affect gene regulation across the genome^[17,18]^. Some PTMs are associated with transcriptional activation (Hostone H3 lysine 27 acetylation (H3K27ac) or Histone H3 lysine 4 trimethylation (H3K4me3)), whereas others mark transcriptional repression (Histone H3 lysine 27 trimethylation (H3K27me3) or Histone H3 lysine 9 trimethylation (H3K9me3))^[19–23]^. Together, these modifications act in combinatorial patterns, often referred to as the histone code, to fine-tune gene regulation. In the heart, such histone-based mechanisms are essential for development, adaptive stress responses, and disease progression^[24–27]^.

(A) Histone Acetylation: This modification involves the addition of an acetyl group to lysine residues on histone tails^[28]^. Histone acetylation generally leads to transcriptional activation by loosening chromatin structure, making DNA more accessible to transcription factors^[28,29]^. Histone acetylation is dynamically regulated in cardiac stress, specifically in cardiac hypertrophy and HF^[28]^. In the heart, p300 [E1A-binding protein p300]-mediated acetylation of histone Histone H3 lysine 27 (H3K27) is critical for transcriptional activation during cardiac development and is required for the expression of the transcription factor GATA-binding protein 4 (GATA4) during heart formation^[30]^. Acetylation of histones Histone H3 lysine 9 (H3K9) and H3K27 has also been implicated in the regulation of GATA4 expression^[31,32]^. Furthermore, global acetylation of histones H3 and H4 exerts protective effects in coronary artery disease^[33,34]^. Abnormal acetylation of histone and non-histone proteins, due to dysregulation of acetyltransferases and deacetylases, is implicated in various human diseases, including cardiometabolic diseases^[35]^.

(B) Histone Methylation: This is a reversible process involving the addition of one, two, or three methyl groups (mono-, di-, or trimethylation) to lysine or arginine residues^[36–38]^. Methyltransferases regulate gene expression through diverse mechanisms beyond the canonical model of site-specific histone methylation. Emerging evidence reveals that individual methyltransferases can target multiple histone residues to exert both activating and repressive effects. Additionally, they modulate transcription *via* methylation of non-histone substrates, including transcription factors and chromatin remodelers, and through methylation-independent interactions within chromatin-associated complexes^[39]^. For instance, histone H3K4 methylation can activate the expression of the Myosin Heavy Chain 6 (*Myh6*) gene in the left ventricle and is generally associated with transcriptionally active genes^[40,41]^. Conversely, H3K9 methylation by Euchromatic histone-lysine N-methyltransferase 1 and 2 (EHMT1/2) is associated with transcriptionally inert heterochromatin and can suppress pathological cardiac hypertrophy^[42]^. The methylation of histone H3K36 plays a dual role in the heart: trimethylation by SET domain containing 2 (SETD2) is essential for normal heart development^[43]^, but demethylation by Nuclear Receptor Binding SET Domain Protein 2 (NSD2) is related to pathological remodeling in cardiac hypertrophy and HF^[44]^. In general, the methylation of histone H3 is highly studied in cardiac development and cardiac disease^[42,43,45]^. However, the methylation of histone H4 is much less studied in the heart, with only one recently published study implicating this modification in different models of cardiac dysfunction^[38]^.

(C) Histone Phosphorylation: This modification involves the addition of phosphate groups to serine, threonine, or tyrosine residues on histone tails^[46,47]^. Histone phosphorylation dynamically modulates chromatin structure and gene expression, influencing chromatin conformation, accessibility, transcription factor binding, and cellular responses to environmental signals. It plays crucial roles in DNA repair, cell cycle regulation, chromosome segregation, and apoptotic responses^[46,47]^. In the adult mouse heart, S10 phosphorylation at histone H3 is strongly increased during the early phase of cardiac hypertrophy and remains detectable during cardiac decompensation, correlating with upregulation of Calcium/Calmodulin Dependent Protein Kinase II Delta (CaMKIIδ) and increased expression of transcriptional drivers of pathological cardiac hypertrophy and fetal cardiac genes^[48]^.

(D) Histone Ubiquitination: Histone ubiquitination is a dynamic PTM that regulates transcription, DNA damage/repair, and chromatin structure. This modification predominantly involves monoubiquitination of histone Histone H2A (H2A) at lysine 119 and histone Histone 2B (H2B) at lysine 120^[49,50]^. Histone H2A lysine 119 ubiquitination (H2AK119ub) mediates transcriptional repression through modulation of the Polycomb complex activity, whereas Ubiquitinated histone H2B at lysine 120 (H2BK120ub) promotes transcriptional elongation and facilitates activating histone marks. In cardiomyocytes, histone ubiquitination regulates stress-responsive gene expression, genome stability, and pathological remodeling. Dysregulation of ubiquitin ligases and deubiquitinases alters chromatin accessibility and transcriptional programs during HF^[51,52]^.

Enzymes (HATs, HDACs, HMTs, and HDMs) involved in histone modifications are dynamically regulated by opposing enzyme classes including:

(A) Histone Acetyltransferases (HATs): These writer enzymes add acetyl groups to lysine residues^[53]^. HATs, such as p300, are crucial for gene activation and play a significant role in epigenetic remodeling in cardiac hypertrophy and HF^[28,30]^. Type A HATs are primarily nuclear and acetylate histones and other chromatin-associated proteins, directly influencing transcription, whereas type B HATs are predominantly cytoplasmic and facilitate the acetylation of newly synthesized histones for nucleosome assembly^[35]^.

(B) Histone Deacetylases (HDACs): These eraser enzymes remove acetyl groups from histone proteins, leading to decreased gene transcription^[53]^. HDACs regulate cardiac plasticity^[54]^ and are critical mediators of inflammation in CVD^[55]^. HDACs 1–3 are important regulators of cardiomyocyte proliferation and function^[54]^. In pathological cardiac remodeling, Class I HDACs (HDACs 1–3) increase the expression of hypertrophic genes, whereas Class IIa HDACs (HDACs 4 and 5) suppress hypertrophy^[56]^. Inhibitors of HDACs (HDACi) have shown potent anti-inflammatory activity and are being explored as potential treatments for CVDs^[57,58]^.

(C) Histone Methyltransferases (HMTs): These writer enzymes add methyl groups to histones^[17]^. The interplay between cardiac transcription factors and histone lysine methyltransferases is crucial for heart development^[59]^. Aberrant expression or mutations in HMTs during development or in adult life can lead to embryonic lethality, congenital heart diseases, and influence the hearťs response to pathological stresses^[59]^. For example, aberrant trimethylation of histone H3K4 by Lysinemethyltransferase 2D (KMT2D) can lead to Kabuki syndrome^[60]^. Similarly, loss of DOT1 like histone lysine methyltransferase (*Dot1l*) in the mouse heart leads to reduced methylation of histone H3K79, which contributes to the development of dilated cardiomyopathy (CM)^[61]^. Although numerous studies have focused on HMT profiling in the heart^[62]^, none have examined how sex influences their regulatory functions in the heart.

(D) Histone Demethylases (HDMs): These eraser enzymes remove methyl groups from histones^[17]^. Histone Demethylases such as Lysine Demethylase 5 and 6 (KDM5C and KDM6A), both encoded on the X chromosome, play critical roles in cardiac gene regulation through the removal of activating H3K4me3 and repressive H3K27me3 histone marks, respectively^[63,64]^. These demethylases often escape X-inactivation, leading to higher expression in females compared to males. Their Y-linked homologs, Ubiquitously Transcribed Tetratricopeptide Repeat Containing, Y-Linked (UT)Y and Demethylase 5D (KDM5D), exhibit reduced or absent enzymatic activity, creating functional asymmetry between the sexes^[65,66]^. Recent evidence further shows that disruption of the *Uty* locus in hematopoietic cells recapitulates the profibrotic attributes of Y chromosome loss in HF, highlighting its non-redundant role in cardiac pathology^[67]^. This differential expression may contribute to sex-biased epigenetic landscapes in the heart, influencing susceptibility to cardiac dysfunction and remodeling. Although direct evidence in cardiac tissue is still emerging, the interplay between sex chromosomes, sex hormones, and histone demethylation suggests a significant role for these enzymes in shaping sex-specific cardiac outcomes.

### Non-coding RNAs

Non-coding RNAs (ncRNAs) constitute a key epigenetic regulatory layer that modulates cardiac gene expression without altering DNA sequence. In the heart, microRNAs (miRNAs), long ncRNAs (lncRNAs), and circular RNAs regulate cardiomyocyte differentiation, metabolic maturation, stress responses, and pathological remodeling by targeting transcriptional and chromatin-regulatory pathways^[68]^. miRNAs repress gene expression through messenger ribonucleic acid (mRNA) degradation or translational inhibition, whereas lncRNAs regulate transcription by scaffolding chromatin complexes or recruiting epigenetic enzymes^[69]^. Dysregulation of ncRNAs contributes to cardiac hypertrophy, fibrosis, ischemic injury, and HF. NcRNA expression and function are influenced by sex chromosomes and sex hormones, contributing to sex-specific cardiac phenotypes^[70]^.

### Sex-specific epigenetic regulation

Sex hormones modulate DNA methylation patterns through DNMT/TET-dependent mechanisms, contributing to sex-specific transcriptional regulation^[71,72]^. Environmental exposures can also induce sex-specific epigenetic effects. For example, gestational lead exposure in mice results in persistent and sex-dependent DNA methylation changes in adulthood^[73,74]^. In humans, several cardiovascular risks show female-specific associations with DNA methylation, including methylation of the Phospholipase A2 Group VII (*PLA2G7*), Actin Beta (*ACTB*), Insulin (*INS*), and Guanine Nucleotide binding protein, Alpha Stimulating (*GNAS*) loci, which correlate with coronary heart disease or myocardial infarction (MI) risk in women but not men^[75,76]^. In addition, sex hormones are also proposed to influence histone-modifying enzymes. Estrogen and androgen receptors can recruit histone-modifying complexes to target loci, suggesting a mechanism for sex-specific chromatin regulation in the heart. This raises the possibility that HMTs and HDMs function differently in male and female hearts, particularly under stress, although direct experimental evidence remains limited^[77]^.

Sex differences in CVD across the lifespan are traditionally attributed to sex chromosomes and circulating sex hormones, but epigenetic mechanisms are increasingly recognized as important mediators of these effects^[78]^. Although many cardiovascular epigenetic studies have not stratified by sex, emerging data support distinct sex-specific epigenetic landscapes in the heart^[79]^. Sexual dimorphism is evident early in cardiac development, before gonad formation, driven in part by sex chromosome composition (XX *vs*. XY), and these early epigenetic differences can persist into adulthood^[75,79]^. Consequently, when sex hormones become active, they act upon a cardiac epigenome that is already sexually distinct. Sex hormones may further shape these differences by directly regulating chromatin-modifying enzymes. Cofactors such as C-terminal-binding protein (CBP) and p300, which possess intrinsic HAT activity, are recruited by estrogen and androgen receptors, providing a mechanism for sex-specific transcriptional regulation. Additionally, some DNA demethylases, such as those from the Jumonji and AT-Rich Interaction Domain (JARID) family, are encoded on the X and Y chromosomes and are activated by estrogen, potentially leading to different effects between males and females^[80]^.

Sex-dependent differences in HDAC activity influence cardiac remodeling. Cardiomyocyte-specific HDAC overexpression causes early mortality in male but not in female mice, indicating increased male susceptibility to HDAC-mediated transcriptional repression. In HDAC5-overexpressing models, repression of Myocyte Enhancer Factor 2 (MEF2) led to mitochondrial dysfunction and reduced Peroxisome Proliferator-Activated Receptor Gamma Coactivator 1-Alpha (PGC-1α) expression in males, whereas females were protected and maintained mitochondrial integrity. Similarly, during pathological left ventricular hypertrophy, female hearts exhibit less suppression of PGC-1α-dependent metabolic genes than males, suggesting more resilient mitochondrial regulation^[81]^.

**Figure 2.**
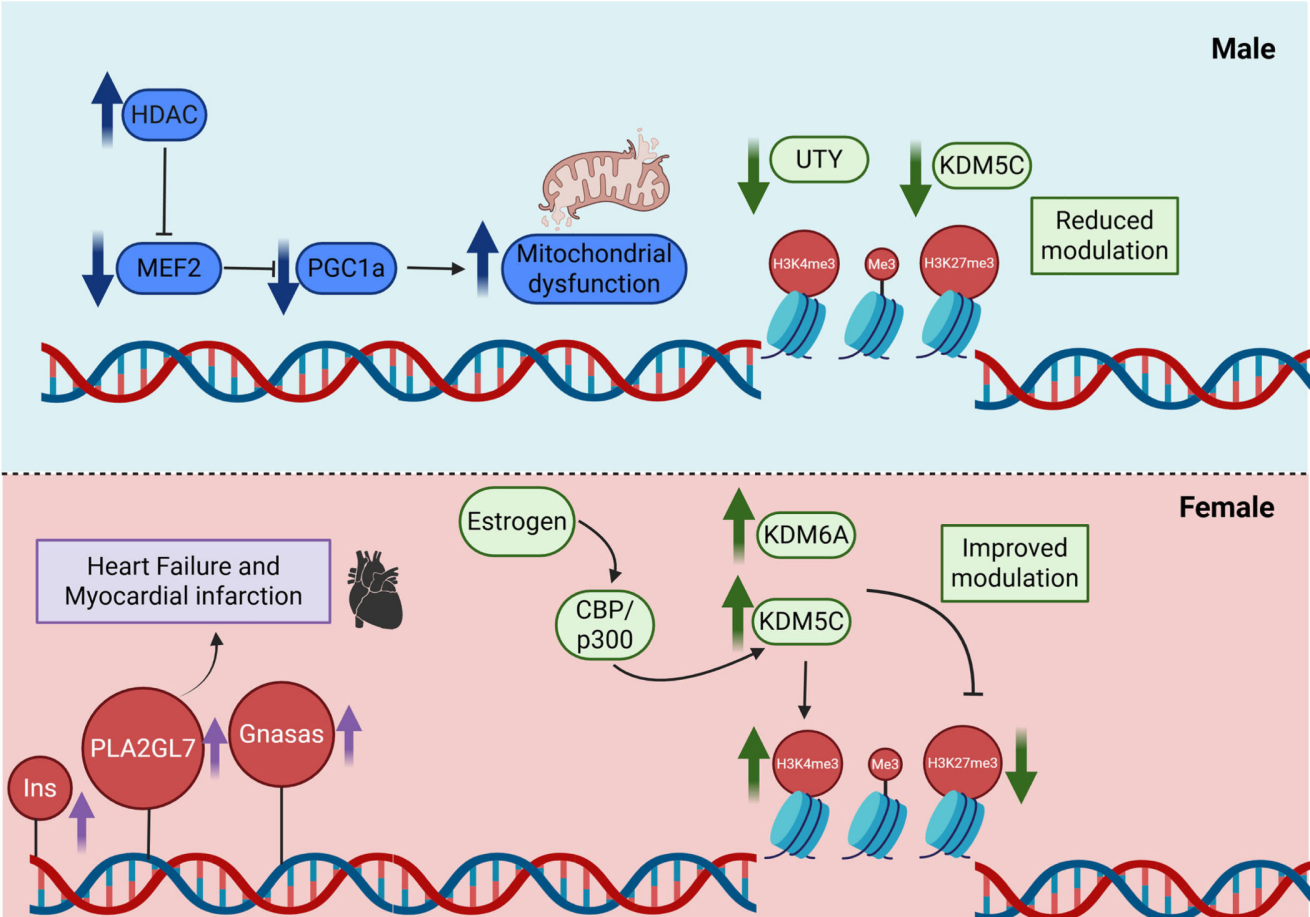
Sex-specific epigenetic regulation of cardiac gene expression. Males exhibit reduced activity of Y-linked histone demethylases (UTY, KDM5D) and greater HDAC-mediated repression of MEF2-PGC1α signaling, contributing to mitochondrial dysfunction and maladaptive remodeling. Androgen signaling may further influence DNMT activity and DNA methylation patterns. Females express higher levels of X-linked demethylases (KDM5C, KDM6A) that escape X-inactivation and benefit from estrogen-mediated recruitment of coactivators such as CBP/p300, enhancing histone acetylation and demethylation. These combined mechanisms underlie differences in cardiac remodeling, heart failure progression, and myocardial infarction risk between men and women. UTY: ubiquitously transcribed tetratricopeptide repeat containing: Y-linked; KDM5D: lysine demethylase 5D; HDAC: histone deacetylase; MEF2: myocyte enhancer factor 2; PGC-1α: peroxisome proliferator-activated receptor gamma coactivator-1 alpha; DNMT: DNA methyltransferase; KDM5C: lysine demethylase 5C; KDM6A (UTX): lysine demethylase 6A; CBP: CREB-binding protein; p300: E1A-binding protein p300. Created in BioRender. Stephens, S. (2026) https://BioRender.com/n26cejf.

Age further amplifies these differences. Female mice display higher HDAC activity than males, which increases with aging, accompanied by elevated expression of profibrotic class I HDACs in older females^[82]^. In contrast, HAT activity and histone H3 acetylation at lysine 9 and 27 do not differ significantly between sexes, implicating deacetylation rather than acetylation as a major driver of sex-specific epigenetic remodeling in the aging heart^[83]^. Collectively, these findings underscore the importance of incorporating sex as a biological variable in cardiovascular epigenetic studies [Figure 2].

## EPIGENETIC REGULATION IN DEVELOPMENTAL PROGRAMMING

### Epigenetic factors controlling cardiac development

In mammals, the heart is the first organ to become functionally active, initiating contractions and blood circulation as early as three weeks after fertilization^[84]^. This early cardiac activity is driven by a tightly regulated network of developmental genes, including NK2 Homeobox 5 (*Nkx2.5*), GATA Binding Protein 4 (*Gata4*), T-Box Transcription Factor 5 (*Tbx5*), and *Myh6*, which coordinate cardiac lineage specification, morphogenesis, and contractile function^[85–87]^. The precise regulation of these genes is governed by multiple epigenetic mechanisms including DNA methylation, chromatin accessibility, chromatin compaction, and higher-order chromatin structure. Together, these processes establish the transcriptional landscape necessary for proper cardiac development and function.

DNA methylation is a key epigenetic mechanism guiding the transition from fetal to adult cardiomyocytes. Disruption of the enzymes responsible for establishing and modifying this epigenetic mark has profound pathogenic effects in cardiac development. Disruption of *de novo* DNA methylation during development profoundly alters cardiomyocyte maturation^[88,89]^, and survival. Likewise, reduced expression of demethylation TET enzymes has been shown to influence cell fate by modulating wingless-related integration site (Wnt) signaling, which is critical for the commitment of neuro-mesodermal progenitors toward neural or mesodermal lineages during early embryogenesis^[90]^. This phenomenon is associated with premature cardiac dysfunction^[91]^.

Chromatin accessibility is influenced by the strategic placement of activating and repressive histone modifications and the arrangement of nucleosomes along the DNA. Studies using Assay for Transposase-Accessible Chromatin using Sequencing (ATAC-Seq) and Chromatin Immunoprecipitation sequencing (ChIP-Seq) have shown that these chromatin features play a central role in enabling transcription factors to access their target regions, thereby reshaping the gene expression programs necessary for cardiomyocyte maturation^[92,93]^. Several chromatin regulators play critical roles in shaping the accessibility landscape required for appropriate cardiac development, including p300, Polycomb Repressive Complex 2 (PRC2), and the SWItch/Sucrose Non-Fermentable (SWI/SNF) complex. PRC2 establishes repressive H3K27me3 marks to maintain cardiac identity by silencing non-cardiac lineage genes^[94]^. SWI/SNF complex facilitates the repositioning of nucleosomes to allow transcription factors access to DNA^[95]^. Perturbations in any of these factors disrupt cardiac gene regulation, often leading to structural heart defects and embryonic lethality^[96–98]^.

Higher-order chromatin structure is primarily shaped by the coordinated activity of CCCTC-Binding Factor (CTCF) and the Cohesin complex, which together mediate long-range chromatin interactions through a mechanism known as loop extrusion^[99]^. This process organizes the genome into Topologically Associating Domains (TADs) that regulate gene expression by facilitating or insulating enhancer-promoter communication. CTCF plays a key role in establishing chromatin architecture early in development. Although its absence does not impair the transition from morula to blastocyst, it becomes essential by the late stage, when its loss results in developmental arrest^[100]^. In the developing heart, CTCF is crucial for coordinating local chromatin interactions that enable the transcriptional programs necessary for proper cardiac lineage specification and morphogenesis^[101]^. Mutations in CTCF binding sites have been shown to disrupt chromatin looping, impairing enhancer-promoter interactions critical for cardiac gene regulation and structural integrity^[102]^. Similarly, the Cohesin complex, comprising core structural components such as Structural Maintenance of Chromosomes (SMC1A, SMC3), and the Double-Strand-Break Repair Protein RAD21, along with regulatory factors such as NIPBL (the Cohesin-loading factor) and STAG2 (a Cohesin subunit variant), is essential for proper cardiac development^[103]^. Mutations in Cohesin complex genes cause Cornelia de Lange syndrome, a multisystem developmental disorder often characterized by severe congenital heart defects^[104]^. Functional studies have shown that *Rad21* depletion disrupts cardiac looping and chamber formation^[105]^, while loss of *Smc3* leads to impaired cardiac function and weakened enhancer-promoter interactions crucial for the regulation of key cardiac developmental genes^[106]^. Furthermore, deficiency in *Stag2* or *Nipbl* results in ventricular septal defects and embryonic lethality^[107]^, highlighting the pivotal role of Cohesin-mediated chromatin architecture in orchestrating the transcriptional programs necessary for heart development.

Importantly, chromatin architecture mediated by CTCF and Cohesin is not static but is influenced by biological sex through both sex chromosome composition and hormonal signaling. Sex-specific differences in CTCF occupancy and loop strength have been reported in multiple tissues and are shaped by X-chromosome dosage, escape from X-chromosome inactivation, and hormone-dependent chromatin remodeling^[108,109]^. Estrogen and androgen receptors can reshape higher-order genome organization by modulating chromatin accessibility and by functionally interacting with architectural proteins, thereby altering enhancer-promoter contacts in a sex-dependent manner^[110,111]^. In the developing heart, such sex-linked differences in three-dimensional genome organization may influence coordinated regulation of metabolic, stress-response, and morphogenetic gene networks, providing an early epigenetic framework for sex-biased cardiac phenotypes later in life^[112]^.

### Epigenetic factors mediating sex differences during cardiac development

Whether chromatin-associated factors contribute to sex differences during cardiac development and, if so, which specific mechanisms are involved, remains largely unexplored^[113]^. A key limitation in current cardiac epigenetics research is the widespread underrepresentation or insufficient analysis of sex as a biological variable^[114]^, despite the fact that several epigenetic regulators are encoded on the sex chromosomes (e.g., KDM6A, KDM5C, and UTY) and may drive sex-biased chromatin states in the developing heart.

Many studies either ignore sex as a factor or fail to adequately consider it in their experimental design. Increasing availability of transcriptomic datasets that incorporate sex as a biological variable has revealed widespread sex-biased gene expression in adult cardiomyocytes, including on autosomal chromosomes. These findings suggest that sex-specific regulatory mechanisms are already active under baseline physiological conditions and may significantly influence cardiac function^[115]^. This is further supported by studies using induced pluripotent stem cells (iPSCs), where the sex of the donor has been shown to affect cardiac lineage specification^[116]^. When Wnt signaling is inhibited to promote cardiomyocyte differentiation, iPSCs with two active X chromosomes preferentially differentiate into cardiomyocytes, whereas those with a single active X chromosome are more likely to give rise to epicardium-derived cells^[116]^.

During early development, even prior to the influence of sex hormones, the presence of either an X or Y chromosome can drive transcriptional differences that shape sex-biased disease susceptibility^[117,118]^. Evidence from preimplantation embryos indicates that these differences extend to metabolic and epigenetic pathways^[119,120]^. For instance, male bovine blastocysts display higher expression of the *de novo* DNMTs (DNMT3A and DNMT3B) ^[121]^ , along with histone methylation-related genes such as HnRNP methyltransferase, S. cerevisiae-like 1 (*HMT1*) and Interleukin Enhancer Binding Factor 3 (*ILF3*), compared with females^[122]^. These transcriptional patterns are accompanied by localized, rather than genome-wide, differences in DNA methylation, including sex-specific methylation of regulatory regions such as those within the Insulin-Like Growth Factor 2 (*IGF2*) gene^[123]^. Such epigenetic dimorphism may influence global transcriptional activity (higher in females in some contexts) and could have lasting developmental and disease-related consequences later in life^[122]^.

In the heart, this transcriptional divergence is also detectable at early developmental stages, both at the transcriptional level, through the identification of sex-biased gene expression, and at the epigenetic level, as evidenced by the dimorphic distribution of histone marks, which has the potential to influence later sex-biased gene expression^[124]^. In their study, Deegan *et al*.^[113]^ conducted an elegant analysis to assign sex to cardiomyocytes using previously published single-cell RNA-sequencing (scRNA-Seq) data from embryonic mouse hearts at 8.5, 9.5-, and 10.5-days post coitum (dpc). This approach yielded a comprehensive list of sex-biased genes across these developmental stages. To investigate which epigenetic factors may contribute to the establishment of sex-specific transcriptional programs during early heart development, we mined the dataset generated by Deegan *et al*.^[124]^ and identified numerous chromatin-associated and epigenetic regulatory genes [Table 1]. Notably, some of these genes exhibit sex-biased expression in a stage-specific manner, whereas others are consistently expressed across multiple stages. The presence of genes such as X Inactive Specific Transcript (*Xist*), XIST Antisense RNA (*Tsix*)), *Ctcf , Kdm5d*, *Kdm6b*, *Kmt2a*, and *Setd1a* (histone methylation/demethylation), *Cbx3*, Protein Arginine Methyltransferase 5 (*Prmt5)* and Retinoblastoma-Binding Protein 7 (*Rbbp7)* (chromatin remodeling and repression), *Uty* (Y-linked demethylase), WD Repeat Domain 5 (*Wdr5)* (H3K4 methylation complex), and SWI/SNF Related BAF Chromatin Remodeling Complex Subunit C2 (*Smarcc2*) strongly suggests that epigenetic mechanisms including chromatin accessibility, DNA methylation, and higher-order chromatin architecture are key drivers of early transcriptional sexual dimorphism. Among these, CTCF deserves special attention. As a key architectural protein, CTCF functions not only as an insulator but also plays a central role in organizing the 3D genome by demarcating TADs and regulating enhancer-promoter interactions^[125]^. Importantly, in other tissues such as the liver, it has been demonstrated that CTCF and Cohesin contribute to sex differences in the transcriptional landscape both directly, through sex-biased chromatin interactions, and indirectly, by shaping 3D genome organization in ways that either facilitate or restrict enhancer-promoter communication in a sex-specific manner^[108]^. Furthermore, CTCF and Cohesin actively shape sex-biased transcriptional landscapes by establishing insulated chromatin loops that enable specific genes to evade X-Chromosome Inactivation (XCI) and resist heterochromatic silencing, thereby reinforcing female-specific gene expression patterns^[126]^. Taken together, these findings suggest that epigenetic regulators such as CTCF and Cohesin may also exert similar sex-specific regulatory roles in the developing heart.

While the epigenetic roles of estrogen and androgen in the heart have been extensively studied and reviewed^[72,75,127]^, our understanding of how sex-specific epigenetic landscapes are initially established during development and prior to hormonal influence remains limited. Fundamental questions, such as when these differences first arise, whether they are driven by sex chromosome dosage, how they influence lineage specification and organogenesis, and whether they predispose to sex-biased cardiac disease phenotypes, are only beginning to be explored^[113]^. Answering these questions will require deliberate inclusion of both male and female samples in experimental designs, as well as the reanalysis of existing scRNA-Seq and epigenomic datasets with sex treated as a critical biological variable. Equally important is the inclusion of sex chromosomes in genomic and epigenomic analyses, as these regions are traditionally excluded due to challenges related to alignment, dosage compensation, and normalization. Incorporating X- and Y-linked genes is essential for uncovering their roles in shaping sex-biased transcriptional networks. Ignoring them risks overlooking key layers of regulation that may be fundamental to understanding sex differences in cardiac development and disease.

### Sexual dimorphism at the intersection of aging, epigenetic remodeling, and clonal hematopoiesis

Aging superimposes an additional layer of complexity through epigenetic drift, characterized by global hypomethylation, focal hypermethylation, redistribution of histone marks, and increased chromatin heterogeneity^[128]^. These changes impair gene regulation, reduce regenerative capacity, and contribute to cardiac dysfunction. Importantly, aging-related epigenetic remodeling reflects not only chronological age but also biological age^[129]^, which captures the cumulative impact of genetic, epigenetic, environmental, and lifestyle factors on tissue function. DNA methylation-based epigenetic clocks have been developed to estimate biological age and reveal interindividual variability in aging trajectories^[130]^, with several studies reporting sex differences in epigenetic age acceleration^[131]^. In general, females tend to exhibit slower biological aging than males, a pattern that aligns with sex-specific differences in epigenetic drift and CVD risk.

Females often experience slower epigenetic aging, likely due to the protective effect of estrogen and dosage of certain X linked genes, delaying the onset of fibrosis, metabolic decline, and oxidative stress. However, menopause accelerates this drift, narrowing the sex gap in CVD risk. In contrast, males tend to show earlier and more linear epigenetic aging, which may contribute to an earlier onset of ischemic events and systolic dysfunction. Environmental exposures further shape these sex-specific trajectories. Diet, air pollution, circadian disruption, and endocrine disrupting chemicals can all remodel chromatin in a sex-dependent fashion, with high fat diet, for instance, inducing stronger pro-inflammatory and fibrotic enhancer remodeling in male mouse hearts, while females may demonstrate greater chromatin plasticity and recovery^[132,133]^. Developmental exposures can reset methylation and histone patterns at hormone receptor and transcription factor loci, reinforcing the importance of the epigenetic exposome, the cumulative environmental impact on the epigenome, within a sex aware framework.

Together with their role in the developing heart, sex differences in cardiac DNA methylation are increasingly recognized, particularly in response to environmental insults. In a mouse model of perinatal lead exposure, males and females exhibited distinct methylation signatures: male biased changes were enriched for pathways regulating cardiac growth, DNA replication, and hematopoiesis, whereas female-biased changes were associated with morphogenetic and chromatin modifying processes^[73]^. These observations support the concept that environmental cues can reprogram the cardiac epigenome along divergent, sex-specific trajectories.

This principle extends beyond the heart into hematopoiesis, where Clonal Hematopoiesis of Indeterminate Potential (CHIP) has emerged as a potent risk factor for atherosclerosis and HF^[91,134−139]^. CHIP involves the expansion of blood cell lineages from a single mutated hematopoietic stem cell, most often driven by mutations in epigenetic regulators such as DNMT3A and TET2, which together account for over half of CHIP carriers with atherosclerotic CVD^[134–137,139,140]^. While both DNMT3A and TET2 are integral to the DNA methylation cycle, recent work demonstrates that, when mutated, they can also exert their pathogenic effects primarily through transcriptional regulation, independent of their catalytic roles in modifying DNA methylation^[141,142]^, adding a new layer of epigenetic regulation to Sex-Differentially Methylated sites (SDMs) in CVD.

Notably, sex modifies CHIP biology: women show a higher prevalence of DNMT3A-mutant CHIP than men across age groups, with the disparity widening in older individuals. TET2-mutant CHIP shows a similar but non-significant trend toward female predominance, while CHIP driven by other genes exhibits a modest but significant female bias^[143]^. One possible explanation for this paradox, higher prevalence of certain CHIP mutations in women yet lower overall atherosclerotic burden, is that female hormonal and epigenetic environments may temper the pro inflammatory transcriptional programs triggered by these mutations, at least until menopause^[144,145]^. Estrogen mediated modulation of immune cell function, differences in chromatin accessibility at inflammatory gene loci, and the higher baseline activity of certain X linked chromatin remodelers could dampen the vascular consequences of clonal expansion. In this context, women may accumulate CHIP clones without experiencing the same magnitude of downstream vascular injury observed in men, though this protective effect may diminish with aging and loss of estrogen, potentially contributing to the narrowing of the sex gap in atherosclerosis risk later in life. These patterns suggest that sex-dependent selective pressures or clonal fitness advantages shape the distribution and clinical impact of CHIP driver mutations. We speculate that these sex-specific patterns in CHIP biology underscore the importance of the immune-cardiovascular interface in SDMs. Mutations in DNMT3A and TET2 not only alter hematopoietic stem cell self-renewal but also reprogram myeloid lineage cells toward pro inflammatory phenotypes that accelerate atherogenesis^[146]^. However, female immune systems may partially buffer these effects through mechanisms such as reduced basal inflammasome activation, a greater propensity for anti-inflammatory (M2-like) macrophage polarization, and estrogen mediated suppression of nuclear factor of kappa light polypeptide gene enhancer in B-cells (NF-κB)-driven transcriptional programs^[147]^. Epigenetic features unique to females, including enhanced accessibility at reparative gene loci and higher activity of certain X linked chromatin remodelers, may further constrain the vascular impact of clonal expansion. This interplay between sex-specific immune programming and CHIP driven hematopoiesis highlights the need to consider both the origin of somatic mutations and the host’s epigenetic context when assessing cardiovascular risk.

**Figure 3.**
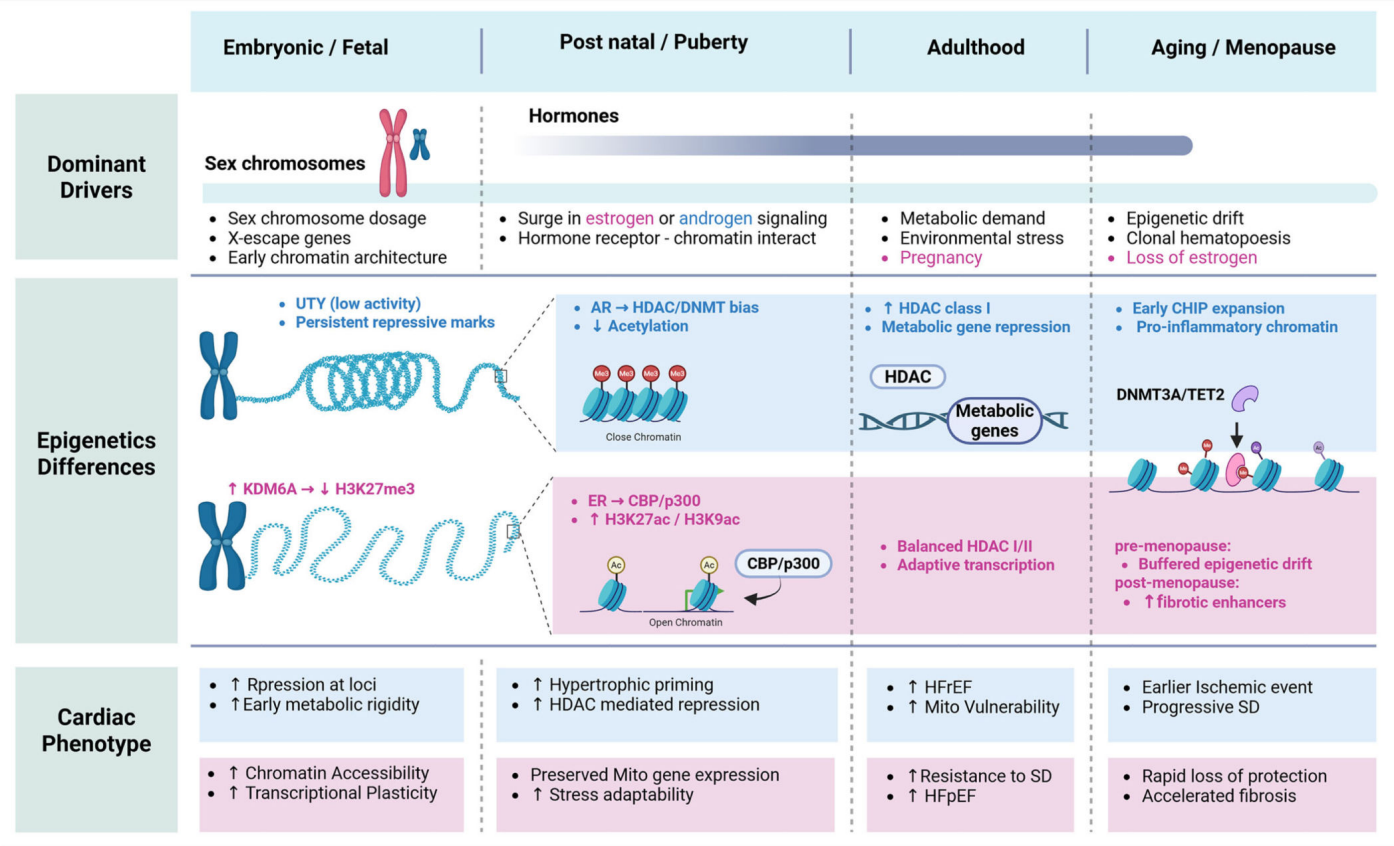
Sex-specific epigenetic regulation of cardiac phenotype across the lifespan. Schematic showing how sex chromosomes, hormones, and aging shape epigenetic regulation and cardiac phenotype from embryonic development through adulthood and menopause. Early sex chromosome dosage establishes divergent chromatin states, with males exhibiting greater repressive marks and females showing increased chromatin accessibility. Pubertal hormone signaling further biases epigenetic regulation, favoring HDAC/DNMT-mediated repression in males and estrogen-dependent histone acetylation and transcriptional plasticity in females. In adulthood and aging, these sex-specific epigenetic trajectories contribute to differential cardiac remodeling, heart failure susceptibility, ischemic risk, and fibrosis. UTY: ubiquitously transcribed tetratricopeptide repeat containing: Y-linked; KDM6A (UTX): lysine demethylase 6A; AR: androgen receptor; ER: estrogen receptor; CBP: CREB-binding protein; p300: E1A-associated protein p300; HDAC: histone deacetylase; DNMT: DNA methyltransferase; TET2: ten-eleven translocation 2; SD: systolic dysfunction; CHIP: clonal hematopoiesis of indeterminate potential. Created in BioRender. Rouzbehani, O. (2026) https://BioRender.com/1ihtyg3.

In summary, sex-specific epigenetic landscapes, shaped by developmental programming, hormonal milieu, environmental exposures, and clonal dynamics in hematopoiesis, profoundly influence cardiovascular aging and disease risk. Differences in DNA methylation patterns, chromatin accessibility, and immune cell programming can modulate the pathogenic potential of somatic driver mutations, such as those in DNMT3A and TET2, in a sex-dependent manner. Recognizing how these intersecting layers of regulation converge on cardiac and vascular health will be essential for developing precision strategies that account for both the genetic origin of mutations and the epigenetic context of the host [Figure 3].

## EPIGENETIC REGULATION IN CARDIAC PATHOPHYSIOLOGY: SEX-SPECIFIC INSIGHTS

### Heart failure (HFpEF, HFrEF)

HF is a heterogeneous syndrome with distinct phenotypes, including HF with preserved ejection fraction (HFpEF) and HF with reduced ejection fraction (HFrEF), both influenced by epigenetic mechanisms such as DNA methylation, histone modifications, and ncRNAs^[148,149]^. Altered DNA methylation programs contribute to pathological remodeling in HF, with phenotype- and sex-specific consequences.

In HFpEF, which disproportionately affects women (prevalence > 60%)^[150,151]^, hypermethylation of promoter regions of genes involved in oxidative metabolism have been observed in left ventricular tissue, leading to reduced expression and impaired energy production^[152]^. This is particularly pronounced in hypoxia-induced models, where DNA hypermethylation promotes cardiac fibrosis, a hallmark of HFpEF^[153]^. In transverse aortic constriction (TAC) mouse model, DNA methylation changes occur in the three days preceding HF. These early changes include hypermethylation of CpG islands at the Matrix Metallopeptidase (*Mmp*)*4* locus, resulting in gene silencing, along with upregulation of Mmp9 that exacerbates cardiac remodeling^[152,154,155]^. Differentially methylated regions (DMRs) have been identified that discriminate HFpEF patients from healthy controls, further supporting a role for DNA methylation in disease. Mutations in DNMT3A and TET2, associated with CHIP, an aging-related risk factor for HFpEF, promotes myocardial inflammation and diastolic dysfunction in mouse models^[91,156,157]^. Additionally, a deep learning model, HFmeRisk, integrates 25 CpG sites with clinical features (e.g., age, BMI, diuretic use) to predict early HFpEF risk with high accuracy (AUC (Area under the curve) = 0.90), linking these methylation sites to pathways such as intercellular signaling and amino acid metabolism^[156]^.

Beyond DNA methylation, HFpEF is associated with alterations in histone modifications and ncRNAs. Reduction in histone modifications, including repressive marks such as H3K9me3 and H3K27me3, are associated with upregulated hypertrophy-related genes^[93]^. In parallel metabolic perturbations reducing nicotinamide adenine dinucleotide (NAD+) availability, impairing sirtuins (SIRT1, SIRT3, SIRT6) activity and deregulating transcriptional programs for fibroblast activation^[158]^. ncRNAs are dysregulated in HFpEF, with panels of miRNAs (e.g., miR-193a, miR-30, miR-106a, miR-191, miR-486, miR-181a, miR-660, miR-199b) implicated in extracellular matrix (ECM) remodeling and fatty acid biosynthesis, showing high power for distinguishing HFpEF from HFrEF ^[159–161]^ . LncRNAs such as *Meg3,* Wisp2 Super-Enhancer-Associated RNA (*Wisper*), Metastasis Associated Lung Adenocarcinoma Transcript 1 (*Malat1*), and *Gata6-as* modulate cardiac fibrosis, while miR-21 promotes fibroblast activation *via* the ERK (extracellular signal-regulated kinase)/MAPK (mitogen-activated protein kinase) pathway^[162,163]^. Circulating miRNAs such as miR-30c, −146a, −221, −328, and −375 serve as biomarkers differentiating HFpEF from HFrEF, with roles in inflammation and fibrosis^[164–166]^.

Sex-specific differences are evident in HFpEF epigenetics, with female hearts showing less downregulation of PGC-1α-dependent metabolic genes, consistent with estrogen-dependent chromatin activation (Section “Non-coding RNAs”) thereby maintaining metabolic gene expression^[30,81,167−170]^. Women exhibit increased ventricular stiffness and fibrotic accumulation in contrast to men, who show more ventricular dilation. This d sex difference is potentially explained by females’ higher type I collagen pro-peptide levels and differential calcium handling. A systematic review by Hartman *et al*.^[78]^ found that only 17% of cardiovascular epigenetic studies stratified data by sex, but those that did, reported higher HDAC activity in females, particularly class I HDACs, which may contribute to increased fibrosis in aging females yet conferring resistance to metabolic stress^[78,171,172]^. In DNA methylation, sex-specific patterns include differential methylation of genes including Olypeptide N-acetylgalactosaminyltransferase 2 (*GALNT2),* Autophagy related 5 (*ATG5*), Ankyrin 2 (*ANK2*), and others, with lead exposures causing sex-dependent changes in pathways such as Notch in males and lysine demethylation in females^[75,173]^. For ncRNAs, sex-biased miRNA networks may drive HFpEF in women, with miR-34a levels decreasing in diabetic women with left ventricular diastolic dysfunction but increasing with progression to HFpEF. Additionally, miR-208b is upregulated more in female failing myocardium, with males showing attenuation *via* Myosin Heavy Chain Associated RNA Transcript (*Mhrt)* methylation and methyl-CpG binding protein 2 (MeCP2)-dependent chromatinization^[174,175]^. X-chromosome escape genes such as *Kdm6a* also regulate sex differences in histone modifications, affecting adiposity and potentially cardiac remodeling. Other studies noted higher levels of Natriuretic Peptides (NPs) and galectin-3, markers of cardiac stretch and fibrosis/inflammation, in women with HFpEF, highlighting sex-specific epigenetic regulation of these pathways^[176,177]^. These findings underscore the need for sex-stratified analyses to understand how epigenetic mechanisms drive differential HF outcomes^[178]^.

HFrEF is more prevalent in men and characterized by systolic dysfunction and cardiomyocyte loss. It is often due to MI or pressure overload, and epigenetic alterations significantly contribute to pathological remodeling^[3,179]^. DNA methylation plays a key role, with hypomethylation of genes such as Matrix Metallopeptidase 2 (*MMP2*) and Connective Tissue Growth Factor (*CTGF*) observed in dilated CM, leading to enhanced collagen turnover and fibrosis^[180–182]^. Knockout of *DNMT3A* in human cardiomyocytes impairs contraction kinetics and alters the MYH7 (Myosin heavy chain 7)/MYH6 (Myosin heavy chain 6) ratio. This leads to lipid vacuole accumulation, and increases susceptibility to metabolic stress, precipitating HF^[183]^. In TAC models, early DNA methylation changes include hypomethylation of Integrin Subunit Alpha 9 (*Itga9*) and Natriuretic Peptide A (*Nppa*) before HF onset, contributing to cardiac hypertrophy^[184]^. Recent studies have identified a 3-markers methylation panel for dilated CM-related HFrEF with excellent diagnostic performance. The genome-wide DNA methylation profiling in end-stage HFrEF reveals metabolic gene reprogramming, including hypermethylation of Tissue Inhibitor of Metalloproteinases 4 (*Timp4*) leading to matrix metallopeptidase 9 (MMP9) upregulation and remodeling^[3]^. Histone modifications are pivotal, with reduced histone H3K4 and H3K9 trimethylation in end-stage non-ischemic dilated CM linked to increased Jumonji Domain-Containing 1C (JMJD1C) expression, hypertrophy and fibrosis. Histone H3 phosphorylation at serine 10 (H3S10ph) is elevated during early hypertrophy and persists in decompensated HFrEF, correlating with CaMKIIδ upregulation and fetal gene reactivation, as observed in both mouse models and human end-stage HFrEF^[48,185]^. Histone acetylation mediated by p300 promotes cardiac hypertrophy, whereas Class II HDACs suppress hypertrophic signaling. Histone ubiquitination defects, such as H2B mono-ubiquitination, link to congenital heart diseases with high HFrEF risk, and H2A ubiquitination contributes to post-ischemic injury^[186,187]^. ncRNAs also play a role, with dysregulated miRNAs such as miR-1, miR-133, and miR-208 associated with cardiomyocyte apoptosis and fibrosis in HFrEF. LncRNAs such as HI19 and Myocardial Infarction Associated Transcript (*Miat*) regulate cardiac remodeling. Circulating miRNAs, including miR-21 and miR-29, are upregulated in HFrEF, modulating inflammation and Extra Cellular Matric (ECM) remodeling^[188–190]^.

Sex differences are notable in HFrEF epigenetics, with males showing higher susceptibility due to androgen-driven DNMT1 expression and hypermethylation of pro-survival genes. Females exhibit higher expression of X-linked histone demethylase KDM6A, which escapes X-inactivation and reduces repressive histone H3K27me3 marks, potentially mitigating severity by enhancing contractile gene expression^[64,72,191]^. Bridges *et al*.^[75]^ reported that KDM6A and other X-escape genes contribute to sex-biased epigenetic landscapes, with females exhibiting a protective profile. Environmental exposures such as lead cause sex-specific DNA methylation changes, affecting Notch pathways in males and lysine demethylation in females, influencing genes such as *Galnt2* and Lysosomal Associated Membrane Protein 2 (*Lamp2*)^[73,173,192]^. Histone modifications show protective effects in females post-myocardial infarction (MI) due to the absence of HDAC5/9, and estrogen alters histone marks to mitigate dysfunction. In contrast, males exhibit increased histone H3K9me3 by EHMT1/2, silencing contractile genes and worsening outcomes^[42,193]^. miR-1 downregulation in males is associated with increased cardiomyocyte apoptosis, potentially due to reduced estrogen-mediated protection. In contrast, sex-biased miRNA networks, such as the preferential upregulation of miR-208b in females, may be attenuated in males through methylation-dependent regulation of the lncRNA Mhrt^[175,194]^. A study by Huo *et al*.^[195]^ highlighted the role of Lysine-Specific Demethylase 1 (LSD1) in HFrEF, with increased expression in human dilated CM and mouse models, particularly in cardiac fibroblasts, suggesting a role in the regulation of fibrosis. Consistent with hormone-dependent chromatin regulation (Section “Non-coding RNAs”), sex-specific stress responses in HFrEF involve differential engagement of acetylation/deacetylation programs^[80,196]^.

### Ischemic heart disease

IHD, encompassing MI and ischemia-reperfusion (I/R) injury, is modulated by epigenetic mechanisms that influence infarct size, inflammation, and repair. Sex-specific differences in these processes are driven by DNA methylation, histone modifications and sex hormone interactions.

Epigenetic remodeling, including altered DNA methylation (Section “DNA methylation”), influences infarct size and repair after ischemic injury. Hypermethylation of CpG islands in pro-inflammatory genes, such as Tumor Necrosis Factor *α* (*Tnf-α*), exacerbates tissue damage during I/R injury, while hypomethylation of antioxidant genes such as Superoxide Dismutase 2 (*Sod2*), regulated by TET enzymes, promotes recovery^[197–200]^. Recent studies highlight the role of mRNA N6-methyladenosine (m6A) methylation in IHD, where m6A writers, erasers, and readers regulate processes such as calcium homeostasis, endothelial function, cell death, autophagy, endoplasmic reticulum stress, macrophage response, and inflammation^[201–203]^. In I/R models, lysine acetylation regulates injury, with HDAC6 deacetylating peroxiredoxin 1 at K197, increasing reactive oxygen species and exacerbating oxidative damage^[204,205]^. SET And MYND Domain Containing 1 (SMYD1), a HMT, was originally identified to trimethylated histone H3K4 (mark of gene activation) and play a critical role in embryonic cardiac development in mice^[206]^. More recent functional studies have demonstrated that SMYD1 protects the heart from ischemic injury by synergistically regulating PGC-1α and Optic Atrophy 1 (OPA1). Through this regulation, SMYD1 helps maintain the expression of electron transport chain subunits and remodeling of cristae structure, thereby enhancing mitochondrial respiration and adenosine triphosphate (ATP) production^[39,207]^. ncRNAs, such as miR-103/107 targeting Fas-Associated protein with Death Domain (FADD) in I/R models and *Hotair* regulating phosphatase and tensin homolog (PTEN) *via* miR-19/29b, contribute to cardiac pathology. Circulating ncRNAs such as *Miat* and Smooth Muscle and Endothelial Cell Enriched Migration/Differentiation-Associated Long Non-Coding RNA (*Sencr*) contribute to MI and diastolic dysfunction^[208–211]^. A study by Kessler *et al*.^[212]^ reported that concentric hypertrophy in females, post-MI, contrasts with eccentric hypertrophy in males, driven by sex-specific epigenetic regulation of hypertrophy pathways.

Sex-specific differences are notable, with hypermethylation of *INS* and *GNAS* loci associated with increased MI risk in women but not in men, suggesting a sex-specific epigenetic predisposition^[75,76]^. In males, androgens increase DNMT1 expression, leading to hypermethylation and repression of pro-survival genes, which may contribute to larger infarct sizes^[71]^. Conversely, estrogen enhances TET2 activity in females, promoting demethylation and upregulation of cardioprotective genes, resulting in smaller infarcts^[77,144,213]^. Sex hormones further modulate ischemic responses through differential engagement of chromatin-modifying enzymes (Section “Non-coding RNAs”)^[80,196,214]^. Females exhibit higher baseline HAT activity due to estrogen-mediated recruitment of CBP and p300, enhancing protective effects such as anti-apoptotic gene activation *via* histone H3K9 (Histone H3 lysine 9)/27ac (27 acetylation)^[30,80]^. In males, increased class I HDAC activity suppresses anti-apoptotic genes, worsening I/R injury^[58]^. Histone methylation, such as H3K4me3 by KMT2D, promotes repair-related gene expression post-I/R, but its dysregulation in males impairs tissue repair^[60]^. Liu *et al*.^[215]^ reported that DOT1L-mediated H3K79 demethylation drives T-box transcription factor 6 (*Tbx6*) expression in cardiomyocytes, promoting stress-induced hypertrophy post-MI, with higher expression in males. Histone phosphorylation, particularly H3S10ph, is elevated during I/R and correlates with pro-inflammatory gene expression, with males showing higher levels than females^[47,216]^. For ncRNAs, sex-biased miRNAs such as miR-21 regulate inflammation, with differential expression contributing to prolonged inflammation in males *via* elevated histone H3K9me3 silencing of anti-inflammatory genes^[42,217]^. A study by Solela *et al*.^[218]^ identified blood-based epigenetic biomarkers, including DMRs in genes such as *PLA2G7*, associated with macrovascular events in women with type 2 diabetes, increasing sex-specific risk profiles. Kessler *et al*.^[212]^ noted that females develop HFpEF post-MI, while males develop HFrEF, driven by sex-specific epigenetic regulation of hypertrophy and inflammation pathways^[212]^. These differences highlight the need for sex-specific therapeutic approaches in IHD management [Table 2].

### Cardiomyopathies

Cardiomyopathies, including hypertrophic cardiomyopathy (HCM), dilated CM, and left ventricular non-compaction (LVNC) involve epigenetic dysregulation that contributes to structural and functional abnormalities, with sex-specific differences influencing disease severity and progression. In HCM, DNA hypomethylation of CTGF and MMP2 promotes pathological hypertrophy and fibrosis^[152]^. Transcriptome and methylome profiling identified novel genes with altered methylation, such as reduced DNMT3 expression contributing to *Myh6* silencing and impaired contractility^[219]^. Trimethylation of histone H3K9 by EHMT1/2 suppresses hypertrophic gene expression, and its loss exacerbates HCM phenotypes^[42]^. Rewiring of 3D chromatin topology orchestrates transcriptional reprogramming in HCM, and bromodomain and extra-terminal domain (BET) inhibitors, such as JQ-1, reduce TAC-induced hypertrophy. ncRNAs such as miR-208b are upregulated in failing myocardium, with LNA (locked nucleic acid)-based knockdown preventing maladaptive remodeling^[220,221]^. LncRNAs such as *Cdkn2b-as1/Anril*, *Hotair*, and *Loc285194/Tusc7* are upregulated in HCM and serve as potential biomarkers^[222,223]^.

Sex-specific differences in HCM include lower prevalence and milder phenotypes in females, potentially due to estrogen-mediated histone H3K27ac by p300 activating antihypertrophic genes^[30]^. Males exhibit higher histone H3K9me3 levels, correlating with increased fibrosis^[42,224]^. Earlier age at presentation and higher penetrance in men, but more severe HF in women, are often linked to complex genotypes and epigenetic modifiers^[225–227]^. *MYH7* mutations show worse prognosis in males for HF endpoints^[228]^. The transcription factor PR domain containing 16 (PRDM16), which exhibits methyltransferase activity, is more prominently linked to LVNC and dilated cardiomyopathy. Prdm16 deficiency in mice leads to age-dependent hypertrophy, fibrosis and cardiac dysfunction^[229–231]^. In humans, PRDM16 deletion causes CM in the context of chromosome 1p36 deletion syndrome^[232]^. *PRDM16* mutation in humans determines sex-specific cardiac metabolism and identifies key metabolic regulators such as Growth Differentiation Factor 7 (GDF7) and X-box Binding Protein 1 (XBP1)^[230,233]^. Moreover, Huo *et al*.^[195]^ found that LSD1 overexpression in cardiac fibroblasts promotes fibrosis in dilated CM, with potential sex differences due to higher LSD1 expression in males.

Dilated CM involves epigenetic changes such that *Dnmt3a* knockout-induced lipid accumulation and impaired contraction kinetics. Aberrant DNA methylation in 195 regions, primarily in dilated CM, with Methyl-CpG Binding Protein 2 (MECP2) overexpression leading to hypertrophy and arrhythmia^[180,183]^. Histone H3K36 trimethylation by SETD2 is essential for normal cardiac development, and its loss contributes to dilated CM^[43]^. Reduced trimethylation of histone H3K4/K9 linked to increased JMJD1C expression promotes hypertrophy and fibrosis^[234]^. NcRNAs such as *H19* and *Miat* regulate remodeling, with nodal lncRNAs controlling cardiomyocyte cell cycle^[235,236]^. As mentioned above, females exhibit higher KDM6A expression, reducing histone H3K27me3 and enhancing contractile gene expression, potentially mitigating dilated CM severity^[64]^. Males show increased histone H3K9me3, silencing contractile genes and worsening outcomes^[42]^. Bridges *et al*.^[75]^ noted that X-escape genes such as KDM6A contribute to sex-biased epigenetic profiles in dilated CM.

**Figure 4.**
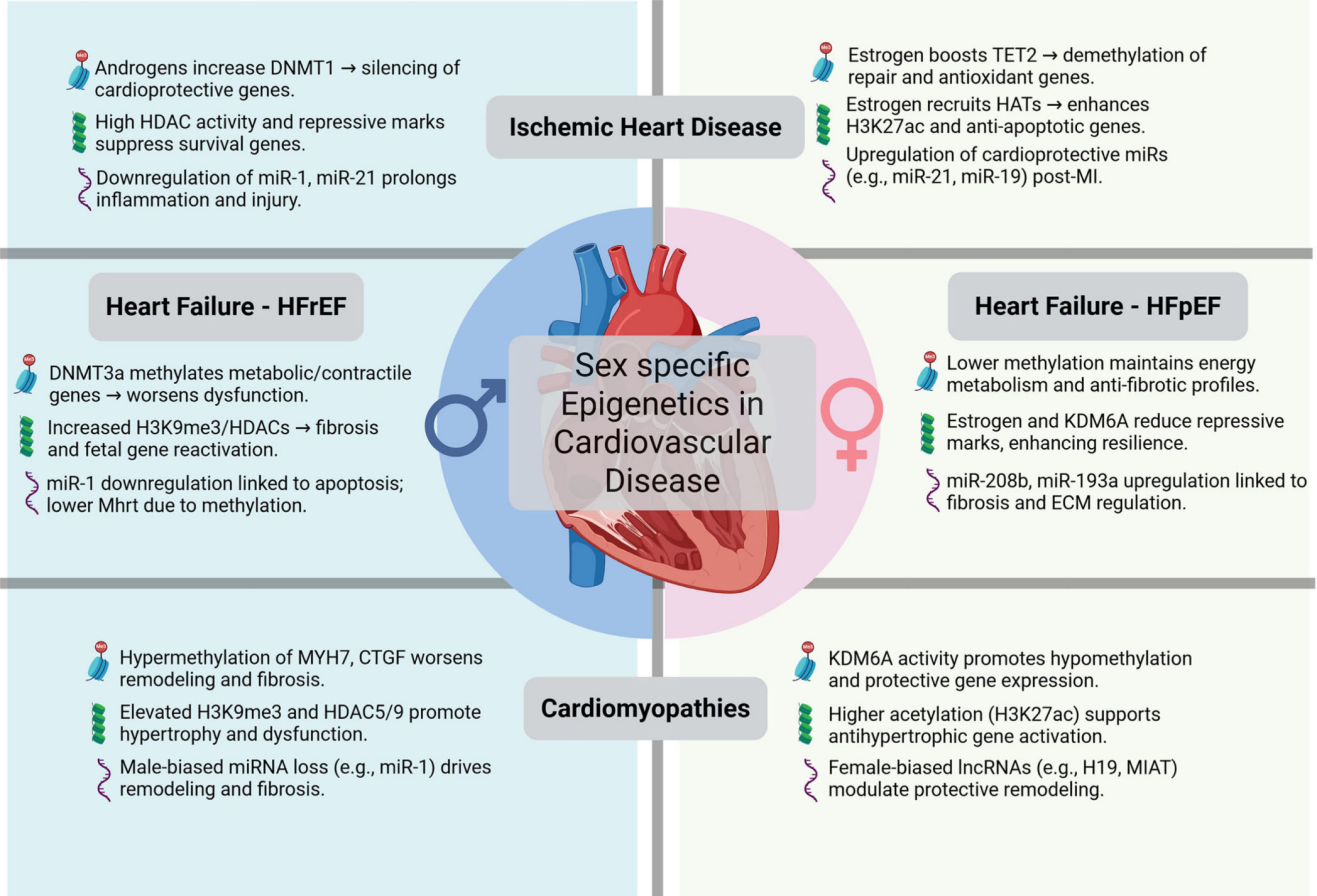
Sex-specific epigenetic regulation in cardiovascular disease. Schematic summarizing epigenetic mechanisms contributing to sex differences in ischemic heart disease, heart failure, and cardiomyopathies. Created in BioRender. Stephens, S. (2026) https://BioRender.com/n26cejf.

LVNC, characterized by excessive trabeculation, is less studied epigenetically. DNA demethylation by TET enzymes is critical for cardiac progenitor differentiation, and its impairment leads to LVNC-like phenotypes^[237]^. Females may benefit from higher TET2 activity, driven by estrogen, promoting trabecular resolution^[77]^. Histone H3K4me3 by KMT2D supports cardiac progenitor gene expression, and its dysregulation may contribute to LVNC, particularly in males with lower HAT activity^[60]^. Radiomics-based classification identifies LVNC *vs*. HCM/dilated CM with high accuracy, highlighting epigenetic-influenced morphological traits^[238–240]^. Transcription factors exhibit sex-specific effects, with mutations leading to differential metabolic regulation and CM outcomes. Further research is needed to fully elucidate these mechanisms [Figure 4].

## THERAPEUTIC IMPLICATIONS

Epigenetic therapies targeting DNA methylation, histone modifications, and ncRNAs offer promising avenues for treating cardiac diseases, with sex-specific considerations critical for optimizing outcomes. Recent studies highlight the potential of these therapies while emphasizing the need for sex-stratified approaches.

DNMT inhibitors, such as 5-azacytidine and decitabine, reverse pathological hypermethylation in HFpEF and IHD, restoring cardioprotective gene expression^[197]^. In HFpEF mouse models, DNMT inhibition reduces fibrosis by demethylating profibrotic gene promoters, with greater efficacy in females due to higher baseline TET activity^[152,241]^. DNMT inhibitors may reduce macrovascular event risk in women with type 2 diabetes by targeting sex-specific DMRs^[242]^. However, off-target effects limit clinical translation, necessitating cardiac-specific delivery systems such as nanoparticles^[243]^.

HDAC inhibitors (HDACi), such as trichostatin A, vorinostat and panobinostat, exhibit anti-inflammatory and antihypertrophic effects in HFrEF and HCM^[58]^. In preclinical models, HDACi suppress class I HDACs, reducing hypertrophic gene expression and improving cardiac function^[57,244,245]^. In addition to effects in cardiomyocytes, HDACi exert actions in cardiac fibroblasts. For example, structurally diverse HDAC inhibitors (TSA, MGCD0103 and apicidin) block cardiac fibroblast cell cycle progression and differentially regulate fibrosis-associated gene expression, likely *via* differential engagement of HDAC1/2 complexes in fibroblasts^[246]^. Importantly, in this study, a subset of fibrotic genes was paradoxically upregulated by class I, selective inhibitor benzamide MGCD0103, highlighting that the anti-fibrotic efficacy of HDACi may depend on inhibitor structure, HDAC complex targeting, and cell type context. Females show greater sensitivity to HDACi due to higher baseline HDAC activity and estrogen-mediated modulation, enhancing PGC-1α expression and mitochondrial function^[58,81]^. In males, HDACi may cause off-target mitochondrial defects, requiring sex-tailored dosing^[81]^. A study suggested that HDACi may mitigate fibrosis in dilated CM by targeting LSD1-associated pathways, with potential sex differences^[247,248]^.

Bromodomain and Extra-Terminal Domain (BET) inhibitors represent a clinically advanced class of epigenetic therapies that target chromatin readers rather than writers or erasers. BET proteins (bromodomain containing 2 (BRD2), bromodomain containing 3 (BRD3), bromodomain containing 4 (BRD4)) bind acetylated histones at enhancers and super-enhancers, facilitating transcription of inflammatory, profibrotic, and stress-response gene programs^[220,249]^. Pharmacological BET inhibition suppresses maladaptive cardiac remodeling by disrupting enhancer-driven transcription without globally silencing gene expression. In preclinical models of HF, pressure overload, and genetic CM, BET inhibitors attenuate pathological hypertrophy, reduce myocardial fibrosis, and improve cardiac function by dampening inflammatory and fibroblast-activated transcriptional networks^[221,250]^. Importantly, BET proteins interact with sex hormone receptors, including estrogen and androgen receptors, providing a mechanistic basis for sex-specific responses to BET inhibition. Estrogen receptor-dependent recruitment of BRD4 to cardioprotective enhancers suggests that BET inhibition may differentially affect transcriptional programs in female versus male hearts, with emerging evidence indicating greater anti-fibrotic efficacy but increased sensitivity to off-target effects in a sex-dependent manner^[251]^. Clinically, the BET inhibitor apabetalone (RVX-208) has been evaluated in cardiovascular outcome trials, demonstrating anti-inflammatory and lipid-modifying effects; however, sex-stratified analyses remain limited, underscoring the need for biologically informed trial design when advancing BET inhibitors for CVD^[252]^.

HMT and HDM inhibitors are emerging therapies. EHMT1/2 inhibitors reduce histone H3K9me3 in HCM, suppressing pathological hypertrophy^[42,253]^. KDM6A inhibitors may balance histone H3K27me3 levels in females, preventing maladaptive remodeling^[64,254]^. Huang *et al*.^[255]^ reported that DOT1L inhibitors reduce histone H3K79me2-driven hypertrophy in MI models, with greater efficacy in males due to higher baseline expression. miRNA-based therapies, such as miR-21 inhibitors, show promise in HFpEF by reducing fibrosis, particularly in females^[256]^.

Sex-specific therapies are critical. Estrogen receptor agonists could enhance HAT activity in females, promoting cardioprotective gene expression, while androgen receptor antagonists in males may reduce DNMT1-driven hypermethylation^[71,257,258]^. Targeting X-linked enzymes such as KDM6A could address sex-biased gene regulation^[65,191]^. Advanced delivery systems, such as viral vectors or nanoparticle-based approaches, are under development to enhance cardiac specificity^[243]^. Clinical trials must incorporate sex-stratified analyses to validate these therapies, as emphasized by Hartman *et al*.^[78]^ and Bridges *et al*.^[75]^.

## CONCLUSION

Epigenetic mechanisms, including DNA methylation, histone modifications, and ncRNAs, are pivotal in regulating cardiac pathophysiology, with sex-specific differences profoundly influencing disease susceptibility, progression, and therapeutic responses. In HFpEF and HFrEF, females exhibit protective epigenetic profiles, driven by estrogen-mediated HAT activity and X-escape genes such as KDM6A, which reduce fibrosis and enhance metabolic gene expression. In IHD, estrogen-driven demethylation and acetylation confer smaller infarct sizes in females, while males show increased inflammation due to androgen-driven hypermethylation and HDAC activity. Cardiomyopathies such as HCM, dilated CM, and LVNC also display sex-specific epigenetic regulation, with females benefiting from protective mechanisms. Emerging therapies targeting DNMTs, HDACs, HMTs, and miRNAs show promise, but sex-specific responses necessitate tailored approaches. The limited stratification of epigenetic data by sex underscores the need for future research to prioritize sex as a biological variable. Integrating these insights into personalized medicine will enhance cardiovascular outcomes for both men and women.

## Figures and Tables

**Table 1.** Sex-biased chromatin and epigenetic regulatory genes in early mouse heart development

Functional category	Male-biased genes	Female-biased genes
Chromatin architecture and 3D genome organization	Cbx3, Cenpa, Ctcf, Hp1bp3, Ino80e, Mau2, Rad17, Smarcal1	-
Histone demethylases (X/Y-linked)	Kdm5d, Uty	Kdm5c, Kdm6a
Histone methyltransferases	Kmt2a, Kmt2e, Nsd1, Set, Setd1a, Setd8, Smyd3	Kmt2d
Histone acetylation and scaffolding factors	Tada3, Taf1, Wdr5	-
Chromatin remodeling (SWI/SNF and associated)	Smarcc2, Arid1b, Cecr2	-
Polycomb & transcriptional repression complexes	Prmt5, Pcgf1, Rbbp7	Lrif1
DNA damage response and chromatin stability	Ercc6, Hltf, Iws1, Jmjd6, Rbm14, Rbm15, Rbm15b	-
RNA-associated chromatin regulators	Elav1, Hnrpc, Hnrpl, Hnrpu	-
X-chromosome regulation and escape genes	Rlim, Taf1	Tsix, Xist
Metabolic/chromatin-linked modifiers	Sirt1, Usp7, Usp22, Spin1, Ythdc1	Meg3

Sex bias reflects consistent enrichment across developmental stages (8.5–10.5 dpc); stage-specific details are provided in Supplementary Table 1. Blue indicates male-biased expression; red indicates female-biased expression.

**Table 2.** Major sex-specific epigenetic mechanisms in cardiovascular disease

Disease context	Dominant epigenetic mechanism	Key sex-specific feature	Functional consequence
HFpEF	DNA methylation, HDAC activity	Estrogen preserves metabolic gene expression in females	Diastolic dysfunction with fibrosis
HFrEF	Histone methylation, HDAC repression	Androgen-associated transcriptional repression in males	Systolic dysfunction and remodeling
Ischemic heart disease	DNA methylation, histone acetylation	Females exhibit enhanced DNA demethylation and repair	Reduced infarct size, improved recovery
Hypertrophic cardiomyopathy	Histone methylation (H3K9me3, H3K27ac)	Females show attenuated hypertrophic signaling	Concentric *vs*. eccentric remodeling
Dilated cardiomyopathy	DNA methylation, chromatin remodeling	X-linked demethylases mitigate severity in females	Progressive dilation and HF
LVNC	DNA demethylation, H3K4 methylation	Estrogen-linked TET activity in females	Trabecular resolution defects

The table summarizes major sex-specific epigenetic mechanisms across cardiovascular disease contexts, highlighting dominant regulatory layers and their functional consequences. Detailed molecular pathways and supporting references are provided in Supplementary Table 2.
